# Profiling *Mycobacterium ulcerans* with *hsp65*

**DOI:** 10.3201/eid1111.050234

**Published:** 2005-11

**Authors:** Sylvia Cardoso Leão, Jorge Luiz Mello Sampaio, Anandi Martin, Juan Carlos Palomino, Françoise Portaels

**Affiliations:** * Universidade Federal de São Paulo, São Paulo, Brazil; † Institute of Tropical Medicine, Antwerp, Belgium

**Keywords:** letter, mycobacterium ulcerans, ulcer, Buruli ulcer, hsp65

**To the Editor:** *Mycobacterium ulcerans* is an emerging human pathogen responsible for Buruli ulcer, a necrotizing skin disease most commonly found in West Africa, but outbreaks have also been reported in the Americas, Australia, and Asia (*1*). Environmental sources of infection and mode of transmission are not completely known. *M. ulcerans* grows slowly at 32°C, requiring 6–8 weeks for colonies to be visible in primary culture. Differentiation from *M. marinum*, which also causes skin infections, is important, since *M. marinum* can usually be treated with antimicrobial agents, whereas *M. ulcerans* most often does not respond favorably to drug therapy, and treatment is usually by surgical excision (*2*). *M. shinshuense*, initially isolated from a child in Japan, is phenotypically and genetically related but biochemically distinct from *M. ulcerans* (*3*).

In the last decade, several DNA-based techniques for mycobacterial identification have been developed. Rapid molecular detection and differentiation of organisms that cause skin infections directly from tissue or exudates could be of great value for early treatment. Some techniques, especially those that include nucleic acid amplification, could be used directly on clinical samples. The accepted standard for molecular identification of mycobacteria is sequencing analysis of 2 hypervariable regions identified in 16S rRNA gene. *M. marinum* and *M. ulcerans* share identical 5´-16S rDNA and 16S-23S rRNA gene spacer sequences (*4*). Polymerase chain reaction (PCR)-dependent methods are based on the 16S rRNA gene (*5*), the *hsp65* gene (*6*) or the insertion sequence IS*2404* (*7*). Recently, a novel category of variable number tandem repeats that could distinguish *M. marinum* and *M. ulcerans* genotypes has been described (*8*).

Polymorphisms in the 3´-16S rDNA region discriminate *M. ulcerans* from *M. marinum* and *M. shinshuense* (*5*). These polymorphisms also allow the separation of *M. ulcerans* into 3 subgroups according to geographic origin and variable phenotypic differences. IS*2404* discriminates *M. ulcerans* from *M. marinum* (*7*). It has been used in restriction fragment length polymorphism analysis applied to a comparable number of *M. ulcerans* and *M. marinum* strains, confirming that this sequence is present in high copy numbers in *M. ulcerans* but absent in *M. marinum*. Nevertheless, an unusual mycobacterium was recently isolated that is closely related to *M. marinum* by phenotypic tests, lipid pattern, and partial 16S rDNA sequencing but presents low copy numbers of this element (*9*).

PCR-restriction enzyme analysis (PRA) of a 441-bp fragment of the *hsp65* gene is a rapid, easy, and inexpensive method for identifying mycobacteria (*10*). Devallois et al. (*6*) described the PRA-*hsp65* pattern of 1 *M. ulcerans* strain ATCC 33728 that originated in Japan. This isolate was considered a new species that resembled *M. ulcerans* and was named *M. shinshuense* (*3*).

We report here the usefulness of PRA-*hsp65* to differentiate *M. ulcerans* strains from different geographic areas. Since Buruli ulcer cases have been reported on 5 continents, we studied 33 *M. ulcerans* strains that originated from Africa (Benin, Zaire, Ghana, Congo, Angola, Côte d'Ivoire, Togo), Asia (China, Malaysia), Australia (Papua New Guinea, Australia), the Caribbean (Mexico, Surinam, French Guiana), 1 *M. shinshuense* from Japan, 1 *M. marinum* isolate and 1 IS*2404*-positive *M. marinum* isolate from France (*9*). All strains were identified at the Institute of Tropical Medicine, the World Health Organization Collaborating Centre for the Diagnosis and Surveillance of *Mycobacterium ulceran*s Infection by IS*2404* PCR and biochemical tests (Table).

**Table:** Origin of strains used in this study*

ITM no.	Species identification	Geographic origin	Source	Providers†	PRA-*hsp65*
960657	*Mycobacterium ulcerans*	Angola	Human	ITM	I
960658	*M. ulcerans*	Angola	Human	ITM	I
5142	*M. ulcerans*	Australia	Human	ATCC 19423	I
5147	*M. ulcerans*	Australia	Human	JS	I
8849	*M. ulcerans*	Australia	Human	DD 8471/69	I
9540	*M. ulcerans*	Australia	Human	DD 11098	I
9550	*M. ulcerans*	Australia	Human	DD 17679	I
940339	*M. ulcerans*	Australia	Human	ITM	I
1441	*M. ulcerans*	Benin	Insect	ITM	I
9146	*M. ulcerans*	Benin	Human	ITM	I
940512	*M. ulcerans*	Benin	Human	ITM	I
940886	*M. ulcerans*	Benin	Human	ITM	I
970010	*M. ulcerans*	Benin	Human	ITM	I
970104	*M. ulcerans*	Benin	Human	ITM	I
970111	*M. ulcerans*	Benin	Human	ITM	I
980912	*M. ulcerans*	China	Human	W.R Faber	II
5150	*M. ulcerans*	D.R.Congo	Human	ITM	I
5151	*M. ulcerans*	D.R.Congo	Human	ITM	I
5155	*M. ulcerans*	D.R.Congo	Human	ITM	I
7922	*M. ulcerans*	French Guiana	Human	IPP 1410900	I
970321	*M. ulcerans*	Ghana	Human	ITM	I
970359	*M. ulcerans*	Ghana	Human	ITM	I
970483	*M. ulcerans*	Ghana	Human	ITM	I
940511	*M. ulcerans*	Côte d'Ivoire	Human	ITM	I
940662	*M. ulcerans*	Côte d'Ivoire	Human	ITM	I
940815	*M. ulcerans*	Côte d'Ivoire	Human	ITM	I
941328	*M. ulcerans*	Malaysia	Human	K. Jackson 18651	I
5114	*M. ulcerans*	Mexico	Human	PL	I
5143	*M. ulcerans*	Mexico	Human	ITM	I
9537	*M. ulcerans*	Papua New Guinea	Human	DD 11878	I
941331	*M. ulcerans*	Papua New Guinea	Human	ITM	I
842	*M. ulcerans*	Surinam	Human	VK 701357	I
970680	*M. ulcerans*	Togo	Human	ITM	I
8756	*M. shinshuense*	Japan	Human	ATCC 33728	II
1027	*M. marinum*	France	Human	VV IPP 99363	I
1026	*M. marinum*	France	Human	VV IPP2000372	I

*PCR restriction enzyme analysis–*hsp65* patterns: I [*Bst*EII and *Hae*III (bp) of 235/210/0 and 145/105/80] and II [*Bst*EII and *Hae*III (bp) of 235/210/0 and 190/105/80]. †ATCC, American Type Culture Collection; ITM, Institute of Tropical Medicine, Antwerp, Belgium; IPP, Institut Pasteur, Paris, France; VK, Academic Medical Center, Amsterdam, the Netherlands; JS, J Standford, School of Pathology, London, United Kingdom; DD, D Dawson, Laboratory of Microbiology and Pathology, Queensland Health, Australia; PL, P Lavalle, Centro Dermatologico Pascua, Mexico City, Mexico; VV, V Vincent, IPP, Paris, France.

DNA extracted from cultures by 3 freeze-boiling cycles was used for amplification, according to the protocol described by Leao et al. (*10*). Gel images were analyzed by using GelCompar II v. 2.5 (AppliedMaths, Sint-Martens-Latem, Belgium). Two distinct *M. ulcerans* PRA-*hsp65* patterns were identified. Of 36 strains, 34 had a PRA-*hsp65* pattern indistinguishable from that of *M. marinum* [*Bst*EII and *Hae*III (bp) of 235/210/0 and 145/105/80] at the Swiss PRAsite (http://app.chuv.ch/prasite/index.html). Two strains, 1 each from Japan and China, showed a different pattern [*Bst*EII and *Hae*III (bp) of 235/210/0 and 190/105/80], that described by Devallois et al. (*6*).

We have shown that PRA-*hsp65* analysis performed on several *M. ulcerans* strains from different geographic areas produced different patterns. In fact, the unique PRA-*hsp65* profile of the *M. ulcerans* strain previously published (*6*) was the most rarely found pattern among the profiles found in this study. This work helps to clarify the PRA-*hsp65* patterns of *M. ulcerans* found in different countries. Because the epidemiology of Buruli ulcer is poorly understood, new molecular tools are still needed to differentiate *M. ulcerans* from different geographic settings, mainly in Africa, where the disease is more prevalent. The PRA-*hsp65* method represents a rapid, easy, and inexpensive technique to differentiate *M. shinshuense* from *M. ulcerans* and *M. marinum*.
